# Non-coding variation in dementias: mechanisms, insights, and challenges

**DOI:** 10.1038/s44400-025-00012-4

**Published:** 2025-06-03

**Authors:** Brianne B. Rogers, J. Nicholas Cochran

**Affiliations:** HudsonAlpha Institute for Biotechnology, Huntsville, AL USA

**Keywords:** Genetics, Dementia, Neurodegeneration, Neurodegenerative diseases

## Abstract

Dementia encompasses many neurodegenerative disorders. While some causal coding variants are known, most GWAS variants are in non-coding regions of the genome, making understanding functional impacts challenging. This review explores the role of non-coding variation in dementia, covering methods to identify enhancers and their target genes, prioritize GWAS variants, and validate the functional effects of variation, providing a comprehensive framework for investigating non-coding variation and its implications in dementia research.

## Introduction

Currently, more than 55 million people have dementia worldwide, with nearly 10 million new cases each year^1^. Dementia can have numerous causes, but typically is the clinical manifestation of underlying pathobiology of neurological death, damage, or dysfunction. In the case of neurodegenerative diseases, the biological processes, such as accumulation of disease-defining proteins (e.g., amyloid-beta plaques, tau tangles, Lewy bodies, etc.) and neuronal loss and/or dysfunction begin long before dementia symptoms like memory loss, confusion, or cognitive decline become apparent. Resultantly, the disease process can take years, or even decades, before passing a pathological threshold to result in noticeable symptoms, making developing treatment targets and determining treatment timing difficult. Therefore, large efforts have sought to identify both biomarkers and genetic contributors to these fatal diseases that may point to novel therapeutic targets that can be used as early interventions, before neurological damage is too severe. Here we focus on genetic contributors to dementias, with a particular focus on non-coding variation, and where our capabilities stand for elucidating the contribution of both common and rare non-coding variation to dementia risk.

## Types of neurodegenerative diseases and genetic contributors

There are many different types of neurodegenerative diseases that result in dementia. These diseases are largely defined by their hallmark pathologies and disease progression through brain regions in a particular order. However, it is often the case that patients present with mixed pathologies, especially in sporadic cases identified in the later decades of life. Here, we discuss major types of neurodegeneration and their hallmark pathologies.

Alzheimer’s disease (AD) is the most common form of dementia, estimated to affect nearly 7 million Americans aged 65 and older^2^. AD is pathologically characterized by the presence of amyloid-beta plaques, neurofibrillary tangles, neuronal loss, and gliosis^3,4^. Symptoms of AD are recognized as a continuum of three broad phases, progressing from preclinical AD to mild cognitive impairment (MCI) to dementia^2,5,6^. At the MCI stage, patients may present with progressive problems with episodic memory, progressing into a clinical diagnosis of dementia as these difficulties interfere with daily activities^6^. AD is classified into two subtypes based on age of onset: early-onset (EOAD; ≤age 65) and late-onset (LOAD, >65 years)^7^. Both forms have a high degree of heritability (90–100% EOAD; 60–80% LOAD)^8,9^. A further subtype of EOAD, autosomal dominant Alzheimer’s disease (ADAD), is caused by rare, deleterious variants that segregate in dominant pedigrees in genes regulating the production and processing of the amyloid-beta peptide (*APP*, *PSEN1*, and *PSEN2*)^10,11^. While age and ApoE allele genotype are the most predominant risk factors for LOAD, there are additional common variants that modulate risk to varying degrees^2,10,12–14^. The most recent and largest LOAD genome-wide association study (GWAS), consisting of over 111,000 clinically diagnosed or proxy AD cases and more than 677,000 cognitively healthy controls, identified 75 genomic risk loci^12^.

Lewy body dementia (LBD) and Parkinson’s disease (PD) are both characterized by the presence of intraneuronal inclusions of alpha-synuclein called Lewy bodies. While they share overlapping symptoms and pathologies, the primary features and progression differ. LBD prominently affects cognition early, leading to memory loss, attention fluctuations, visual hallucinations, and motor symptoms similar to PD^15^. In contrast, PD manifests with motor symptoms like tremors, rigidity, and bradykinesia, with dementia sometimes emerging in later stages of the disease. LBD is diagnosed when cognitive impairment precedes parkinsonian motor signs or begins within 1 year of its onset, whereas in PD, cognitive impairment develops later in disease course and is diagnosed as PD with dementia (PDD). However, both share alpha-synuclein as an underlying pathology. Causative evidence has been established for alpha-synuclein in PD through families with duplication and triplication of *SNCA*, the gene encoding for alpha-synuclein^16,17^. Further efforts to understand *SNCA* transcriptional regulation revealed intronic enhancers of *SNCA* that harbor variants predicted to increase *SNCA* expression^18,19^. Finally, *SNCA* has been associated with both PD and LBD through GWAS. The most recent and largest LBD GWAS analyzed over 2500 individuals diagnosed with LBD and about 4000 healthy controls, identifying five risk loci at *GBA*, *APOE*, *SNCA*, *BIN1*, and *TMEM175*^15^. For PD, Nalls et al. performed the largest meta-GWAS to date, analyzing over 37,000 PD cases, 18,600 proxy-cases (first degree relative with PD), and more than 1.4 million healthy control individuals^20^. This study identified 90 independent risk signals across 78 genomic regions, including multi-signal loci at *GBA*, *NUCKS1/RAB29*, *GAK/TMEM175*, *LRRK2*, and *SNCA*, and novel independent risk variants at *UBTF/GRN* and *FAM171A2*^20^. The Global Parkinson’s Genetics Program (GP2) was recently established to create a worldwide collaborative effort to further our understanding of the genetic architecture of PD by recruiting more diverse individuals and collecting dense genetic data on at least 150,000 participants^21^. This large consortia will allow for more rapid growth of data in PD and help identify more causal variants and genes, including those implicated in non-European ancestries. Recent results of these efforts are currently preprinted^22^.

Frontotemporal dementia (FTD) refers to a diverse group of disorders caused by progressive neuronal loss in frontal or temporal lobes, often occurring before age 65, and divided into three main subtypes^23,24^. Behavioral variant FTD (bvFTD) is the most common form and is characterized by prominent changes in personality and behavior^25^. Primary progressive aphasia (PPA) is the second major form and affects language skills, speaking, writing, and comprehension^26^. Motor dysfunction disorders are also part of the FTD spectrum and can occur with or without bvFTD or PPA, including corticobasal syndrome and progressive supranuclear palsy (PSP)^24^. Pathologically, there are three major groups classified by characteristic patterns of abnormal protein deposition: tau (36–50% of cases), TDP (~50% of cases), and FUS (~10% of cases)^23,27^. The only known risk factors are age and genetics: autosomal dominant mutations in *C9orf72*, *MAPT*, and *GRN* account for about one-third of all cases^23,27^. However, the majority of sporadic FTD cases cannot yet be explained genetically. The largest FTD GWAS analyzed 4685 sporadic FTD cases and 15,308 controls, identifying the *MAPT*, *APOE*, and *RPSA-MOBP* loci as contributing to genetic risk for FTD^28^.

There is a large overlap between amyotrophic lateral sclerosis (ALS) and FTD, and there is evidence that these two diseases form a broad neurodegenerative continuum^29^. ALS is primarily a motor neuron disease, affecting both peripheral motor neurons as well as varying levels of central involvement in the frontal cortex, brainstem, and spinal cord^30^. Typically, symptoms begin as muscle weakness in a limb, or occasionally changes in voice or difficulty swallowing, progressing to generalized weakness and paralysis of respiratory muscles^29^. Cross-sectional studies estimate that up to 50% of ALS patients will develop cognitive impairment associated with FTD, and up to 30% of FTD patients will develop motor dysfunction, diagnosed as FTD-motor-neuron disease (FTD-MND) or FTD-ALS^28,29^. In a recent large-scale genome-wide multitrait association study, Chen et al. evaluated the shared genetic etiology of ALS and FTD. This study found a significantly high genetic correlation (*r*g = 0.637) between the two diseases, as well as causal variants for both ALS and FTD at 9p21.2 (*MOB3B*, *IFNK*, and *C9orf72*) and 19p13.11 (*UNC13A*), which had previously been identified as overlapping disease-associated regions^31,32^. The top variant at the 19p13.11 locus, rs12608932, is located within intron 20 of *UNC13A* and thought to be included in the transcript when TDP-43 levels are reduced, increasing the risk of developing ALS and FTD. Other studies have linked this variant to ALS and suggested that this variant may exacerbate FTD in sporadic ALS cases^31,33^.

Overall, for all types of dementia, there is heterogeneity with symptoms, age of onset, pathologies, and, importantly, genetics. While there are rare variants resulting in autosomal dominant inheritance patterns, many disease-associated variants are common in the population and modify disease risk to varying degrees. The majority of all GWAS nominated variants and ~95% of high confidence fine-mapped single-nucleotide variants (SNVs) are in non-coding and flanking regions, suggesting their possible relevance for disease pathogenesis and as potential therapeutic targets^34–36^. The current leading hypothesis is that these regions are regulatory elements that, when harboring a variant, have altered abilities to contribute to gene expression^37–39^. There may be many variants in a locus that are candidates for contributing to the GWAS signal, and LD can make disentangling the causative variants difficult. For GWAS loci consisting of top statistical candidate variants only in non-coding regions, identifying a top candidate variant can be particularly difficult due to the absence of annotations that often would lead to a clear top candidate, such as if there were a coding variant of high impact. Additionally, linking non-coding variants to target genes is difficult due to the complexity and non-linearity in DNA conformation, which allows regulatory elements to interact with distant targets. Understanding the functional roles of these non-coding regions can help us determine the contribution of variants in these regions to disease onset and progression. We discuss the current state of our ability to elucidate non-coding variant function in the following sections.

## Enhancers

Gene regulatory elements allow for transcriptional control in cell type, temporal, and environmentally responsive manners. There are several types of gene regulatory elements, including promoters, enhancers, silencers, and insulators. It is estimated that the human genome harbors hundreds of thousands to millions of enhancers in its non-coding regions, and it is recognized that a major component of heritability of many common diseases partitions to regions with enhancer-like signatures^39–41^. Therefore, while all of the mentioned classes of regulatory elements serve an important function, we will focus on the role of enhancers for this review, but with many of the points discussed likely applicable to other classes of regulatory elements as well.

Enhancers have been classically defined as *cis*-acting DNA sequences that can increase the transcription of genes^41^. Over the past forty years, since the coining of the term enhancer, many key characteristics have been discovered to help define and identify enhancer sequences. Enhancers are short, non-coding segments of DNA (usually 100–1000 bp) that can control gene expression in a typically orientation-independent manner, and they are distally located from their target promoters, often relatively proximally, but in some cases up to hundreds of kilobases or even 1 Mb away^41,42^. Active enhancers are characterized by bidirectional transcription to produce enhancer RNAs (eRNAs). This active transcription is thought to allow the enhancer locus to remain in a more accessible and active state^39^. Additionally, eRNAs are generally not polyadenylated, making them less stable, and their transient expression allows them to fine-tune expression during development and in response to stress^43^. eRNAs are understudied in dementias, providing an opportunity for future work. The few studies considering the role of eRNAs in neurodegeneration have been recently reviewed elsewhere^44^. Finally, enhancers act in a temporal- and spatial-specific manner. Some enhancers are only active during specific developmental stages and have been associated with critical tissue-specific biological processes, like regulating cell type and condition-specific 3′ UTR isoform expression, which influences mRNA stability, translation, and localization^45^. Many enhancers are also cell type-specific, and within the brain, the enhancer repertoire is different between neuronal and glial cell types^39,40^.

Throughout evolution, variations in the non-coding regions of the genome, like enhancer regions, are thought to play a key role in shaping species-specific traits^46^. Enhancers have undergone rapid sequence turnover through mammalian evolution, and both highly conserved and evolving enhancer sequences have been found to significantly impact phenotypic traits^47^. Highly conserved enhancers tend to regulate fundamental processes, such as embryonic development, and have lower cell type-specificity^47–49^. Sequence divergent, or newly evolved, enhancers are important in establishing tissue and species-specific traits and are often mediated by de novo insertion of transposable elements (TEs) carrying clusters of transcription-factor-binding sites^50–52^. These DNA motifs are highly conserved, despite the divergence of enhancer sequences, suggesting the presence of a genomic regulatory framework that is both conserved and adaptable^53,54^. Importantly, sequence conservation alone does not indicate functionality of an enhancer or reveal information about the cell- and tissue-specific activity of an enhancer^54^.

**Fig. 1:**
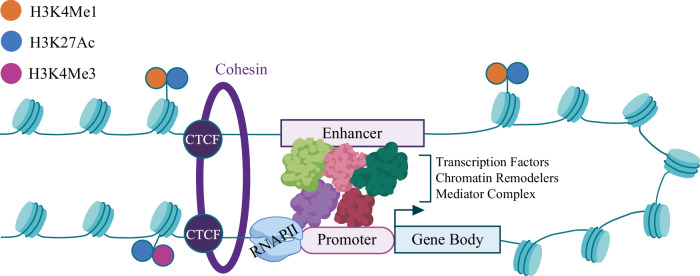
Regulation of gene expression mediated by enhancers. Illustration of chromosomal looping, mediated by CTCF and cohesin, that allows a distal enhancer to be in close proximity and interact with the promoter of its target gene. These interactions increase binding of transcription factors, chromatin remodelers, and the Mediator complex to recruit RNA polymerase II (RNAPII) at the promoter of the target gene. Active promoters and enhancers are in regions of open chromatin, depleted of nucleosomes. Active enhancers are flanked by nucleosomes with specific histone modifications (H3K4Me1 and H3K27Ac).

Many assays have been developed to measure biochemical annotations that correlate with enhancer activity on a genome-wide scale. These include assays for histone modifications and transcription factor (TF) binding (chromatin immunoprecipitation sequencing [ChIP-seq]), chromatin accessibility (DNase-seq or assay for transposase-accessible chromatin [ATAC-seq]), DNA methylation (EM-seq or bisulfite sequencing), and transcriptional activity (RNA-seq)^40^. Data from these assays has provided a basis for a few general principles to help identify enhancers, modeled in Fig. 1. First, enhancers are found in regions free of nucleosomes and therefore transcriptionally accessible. However, the nucleosomes flanking active enhancer regions have specific, transcription-associated histone modifications, like histone 3 lysine 4 monomethylation (H3K4me1) and H3K27 acetylation (H3K27Ac). Poised enhancers often exhibit H3K27Me3, which is associated with the Polycomb repressive complex and is replaced by H3K27Ac for activation^39,55^. Second, enhancers typically contain clusters of transcription factor (TF) binding motifs. The binding of these TFs forms a molecular bridge by tethering enhancers to target gene promoters. Lastly, enhancers can act at varying distances. ENCODE has annotated regulatory elements based on distance from a GENCODE annotated transcription start site (TSS): promoter-like sequences (PLSs) fall within 200 bp center to center of a TSS, proximal enhancer-like sequences (pELSs) are within 2 kb of a TSS, and distal enhancer-like sequences (dELSs) encompass all enhancers beyond 2 kb of a TSS^56^. Very distal enhancers have been identified up to a megabase in linear distance away from their target promoter^57,58^. Overcoming this distance to physically interact with promoters is accomplished through chromatin looping, which is mediated by CTCF and cohesin, allowing enhancers to skip over the nearest gene in linear space to directly interact with the target promoter.

Promoters are thought to contact an average of four to five enhancers; thus, each enhancer is only responsible for a portion of the expression of its target gene^41,55^. This enhancer redundancy provides an extra safeguard against perturbation or variation within enhancers to preserve gene expression. Several studies have shown that enhancer redundancy is common within the human genome, particularly in association with developmental and disease-associated genes^59–61^. Therefore, variants falling in enhancer regions are predicted to affect disease through small effects, and it may be the interaction of multiple variants that pushes the burden past the threshold to present phenotypically as disease.

## Prioritizing non-coding variants

Genome-wide association studies comparing clinically diagnosed individuals to non-demented age-matched controls have been used to identify genetic variation associated with disease risk. Briefly, GWAS uses genetic information, historically usually obtained through microarray genotyping and imputation for economy of scale, to test the association of a single trait with a large number of common SNVs across the genome^62^. In addition to the majority of GWAS variants being in non-coding regions, the genotyped SNVs identified actually represent a group of genetic variants often inherited together as a haplotype block of high linkage disequilibrium (LD). Therefore, SNVs identified in GWAS are not necessarily the causative variant but rather tag a region of the genome that is associated with disease^62^. Therefore, identifying causal variants is challenging, and many methods have been developed to prioritize variants in LD blocks associated with disease.

Fine-mapping strategies (e.g., FINEMAP^63^, PAINTOR^64^, and CAVIAR^65^) attempt to determine the causal variant (or variants) for complex traits, like dementia, given the association of the genomic region with a trait. These methods estimate the posterior probabilities of causality for variants while accounting for LD. Methods like PAINTOR leverage enrichments in functional genomic annotations, often using computational predictors like PolyFun^66^ and EMS^67^, to improve causal variant identification by giving higher weights to variants residing in certain functional annotations^64,68^. Schwartzentruber et al. recently applied fine-mapping techniques, including FINEMAP and PAINTOR, to AD GWAS by proxy meta-analysis data. In this study, 21 variants were identified with causal probabilities above 50% across the different fine-mapping techniques, including SNVs near known AD-risk genes: *BIN1*, *ABCA7*, *NCK2*, *APH1B*, *ADAMTS1*, and *SPRED2*^68^. While these methods have improved significantly over the past decade, challenges like cell type-specificity of enhancer function and lack of relevant cell type datasets of functional genomic annotations have limited their ability to reliably predict causal variants.

Deep-learning models have also emerged as useful methods for prioritizing causal non-coding variants based on their predicted regulatory impact. Methods like DeepSEA^69^ use DNA sequence as input and predict changes in chromatin accessibility and TF binding at single-nucleotide resolution. Newer approaches like ChromBPNet^70^ build on this idea and use single-cell ATAC-seq data to predict high-effect variants in cases compared to controls. Briefly, ChromBPNet uses convolutional neural networks to predict pseudo-bulk cell type-specific chromatin accessibility profiles for scATAC-seq derived cell types from genomic sequence, then cluster-specific models are used to compute local disruption scores based on allelic fold-change in predicted counts for chromatin accessibility^71^. Additional methods combine tissue-specific variant scoring techniques for disease-specific variant prioritization, such as Liang et al. ^72^. Using the NHGRI-EBI GWAS Catalog, this study compiled a dataset of 25,000 non-coding SNVs across 111 disease phenotypes by combining published tissue-specific scores and using a logistic regression approach to derive disease-specific combination weights to associate SNVs with disease^72^. These methods are valuable for identifying cell type- and disease-specific enrichment of non-coding variants and TF motif disruption. However, their effectiveness is constrained by data availability, as less common forms of dementia often have smaller sample sizes of diagnosed individuals, and functional genomics data for relevant cell types in the CNS is growing but is frequently lacking, limiting the ability to analyze variants in critical cell populations.

Rare variants have the potential to exhibit larger effect sizes than common variants because they can have a more deleterious effect on protein function^73^. However, much of the work to understand the role of rare variants in disease has focused on coding regions. Expanding analysis of rare variants to non-coding regions has been challenging due to the large number identified in whole genome sequencing and the likelihood that most have no functional impact. Methods used for common variants, like GWAS, are often underpowered for rare variants due to the sparsity (inherent in their rarity) and high multiple testing burden due to the vast number of rare variants compared to common variants^74^. Current approaches use functional annotations to prioritize variants, such as predicted *cis*-regulatory elements (CREs), especially enhancers and promoters^75^. Often, rare variants are grouped by nearest gene in an effort to increase power, but at the expense of base pair resolution^76^. Despite the low power to pinpoint a single causal variant, rare variant-nominated disease genes have significantly improved our understanding of disease mechanisms. For example, Holstege et al.^77^ compared gene-based burden of rare damaging variants in exome sequencing data and observed significant associations of variants in *ATP8B4* and *ABCA1* with AD risk and a suggestive risk signal in *ADAM10,* in addition to confirming known rare variant burden signals for *ABCA7*, *SORL1*, and *TREM2*. Additionally, they were able to identify *RIN3*, *CLU*, *ZCWPW1*, and *ACE* as potential drivers of respective AD GWAS loci^77^. Therefore, despite the loss of information for individual SNVs, these analyses are still critical for advancing therapeutic strategies. Recently, Das et al.^74^ developed a Bayesian generalized linear model, Genome-wide Rare Variant EnRichment Evaluation (gruyere) to model cell type-specific, non-coding rare variant associations on a genome-wide scale. Here, a variant’s effect is a deterministic function of its functional annotations and the estimated AD-relevance of the gene where the variant is mapped. In this study, they leveraged whole genome sequencing data from the Alzheimer’s disease sequencing project (ADSP) and identified four significant genes: *C9orf78*, *MAF1*, *NUP93*, and *GALNT9*. This model also iteratively learned the importance of functional annotations to AD and determined that splicing, TF binding, and chromatin state were the most enriched for AD-associated non-coding rare variants^74^. Other groups have implemented sequence kernel association testing (SKAT)^78^ for burden analysis of non-coding regions. For example, we utilized SKAT to assess variants identified in whole genome sequencing from 683 individuals with either EOAD or FTD and 856 healthy adult controls in an effort to identify genetic pleiotropy between EOAD and FTD^79^. Cooper-Knock et al. ^80^ took a similar approach in a cohort of 4495 ALS cases and 1925 controls obtained from Project MinE^81^. In this study, they were able to establish *CAV1* as an ALS risk gene, whereas previously it was unclear whether *CAV1* dysfunction was a cause or effect of neuronal toxicity in ALS^80^.

## Enhancer-gene pair identification

While GWAS have advanced our understanding of the genetic architecture of dementias and nominated variants in loci associated with disease phenotypes, functional genomics assays are required to prioritize genes that modulate this disease susceptibility and determine candidate causal genes for further functional validation. These disease-associated variants identified in GWAS are typically assigned to the gene in closest linear distance, which due to DNA looping may not be the gene in closest three-dimensional space or the target of the regulatory region harboring the variant^82–85^. Additionally, disease-associated variants often only function in specific cell types^86,87^. To overcome these challenges, many methods have been developed with the goal of coupling candidate CREs (cCREs) with their respective target genes, taking into consideration the cell type-specificity of these interactions.

Advancements in single cell or single nucleus sequencing have allowed profiling of gene expression and chromatin accessibility in specific cell types, either separately or in parallel from the same samples^86,88–92^. These studies have nominated both cell type-specific transcriptional and epigenetic differences between individuals with neurodegenerative disease and unaffected controls. Integration of single-nucleus ATAC-seq (snATAC-seq) and single-nucleus RNA-seq (snRNA-seq) data allows for the identification of candidate CREs by correlating chromatin accessibility with nearby gene expression. Therefore, relationships between ATAC-seq peaks (cCRE regions) and target genes can be inferred, nominating cCRE-gene target pairs in cell type and disease-specific manners. Different computational methods (described below) allow for CRE-gene pairs to be established using datasets where snRNA-seq and snATAC-seq have been performed separately on different samples of nuclei. However, performing these assays simultaneously in the same nuclei allows for greater confidence in these CRE-gene pair correlations^88^. For example, in our recent study, we employed snRNA- + snATAC-seq in seven AD patients and eight age-matched cognitively healthy controls to identify five times as many CRE-gene links (over 300,000) than previously reported, with an average of two genes linked to each peak and a range of 1–21 linked genes^88^. Using stratified linkage disequilibrium score (sLDSC) regression, we found that AD-specific linked peaks identified in microglia were significantly enriched for heritability of AD, suggesting that variants within these peaks could have a greater contribution to AD risk^88^. Recently, Rexach et al.^93^ applied this same approach to analyze 40 participants with AD, FTD, or PSP. By profiling multiple regions with differential vulnerability across multiple diseases, they were able to show that known risk genes act in specific neuronal and glial states or cell types that differ across related disorders, primarily implicating non-neuronal cells in AD and specific neurons in FTD and PSP^93^. Further, this study found that expression of *RORB*, *KCNH7*, *PDE1C*, *NLGN1*, and *OPCML* was enriched in depleted excitatory neurons of multiple classes across all three disorders, likely contributing to the molecular basis of the selective neuronal vulnerability of these neurodegenerative diseases^93^.

The genome is organized in three-dimensional (3D) space through intricate folding within the nucleus. This 3D arrangement is driven by higher-order chromatin structures, such as topologically associating domains (TADs), chromatin loops, and nuclear compartments, which bring distant regulatory elements, like enhancers and promoters, into close proximity. These spatial interactions enable precise control of gene expression. These long-range interactions are particularly important in complex tissues like the brain, where genes with extended intergenic DNA are enriched compared to non-neural tissues^94^. Because DNA looping allows for regulatory elements to act on promoters of genes at very large distances in linear space, assigning CRE-gene pairs can be difficult. Therefore, many techniques have been developed to understand the spatial organization of chromatin. Molecular assays based on chromosome conformation capture (3C) output genomic sequences, connecting chromosome structure to genomic sequence interactions^95^. Generally, this technique and its derivatives measure the frequency at which two DNA fragments physically associate in three-dimensional space, based on the propensity for those two locations to become formaldehyde-crosslinked together^96^. One such derivative, HiC, allows for “all-versus-all” interaction profiling, allowing for overall genome structure analysis and identification of more specific long-range contacts between distant loci, like CRE-gene pairs^96^. Using these data, studies have been able to show interactions through HiC loops of GWAS SNVs with target gene promoters, nominating causal genes for disease-associated signals^97^. One limitation of HiC has been the sequencing depth required to obtain high-resolution maps required to identify CRE-gene pairs with high confidence. Therefore, derivative methods using the “one-versus-many” or “many-versus-many” approaches have still gained traction to reduce sequencing costs. For example, Corces et al. generated chromatin interaction maps using HiChIP for H3K27ac, which marks active promoters and enhancers^86,98^. Combining this data with single-cell ATAC-seq data identified multiple loci with microglia-specific accessibility and nominated putative causal genes affected by AD-risk variants^86^. In a recent publication, we utilized Capture-C to identify CREs interacting with the *MAPT* promoter in human differentiated glutamatergic and GABAergic neurons^99^. Briefly, this method uses biotinylated RNA oligomers complementary to the target loci (here the *MAPT* promoter), revealing long-range interacting regions that contain enhancers, silencers, or other promoters^100^. By utilizing this method, we identified 13 cCREs interacting with the *MAPT* promoter not identified by HiC within the same cell type^99^.

As functional genomics assays advance, the computational methods used to analyze these large datasets are continuously evolving. Many groups have built computational models to predict enhancer-gene regulatory interactions. Examples like the activity-by-contact (ABC)^101^ model and ENCODE-rE2G^102^ utilize bulk measurements of chromatin accessibility and 3D contacts. The ABC model estimates enhancer effects by multiplying enhancer activity (ATAC-seq and H3K27ac ChIP-seq reads) by the 3D contact frequency, and then dividing this product by the sum of the products of all candidate enhancer activities and their respective contact frequencies to calculate a relative contribution for each enhancer^101^. Another recently developed package is DegCre, a nonparametric method that estimates an association probability for each possible pair of enhancer and target gene under the assumption that both should change in concert with one another as a result of a perturbation^103^. This method uses rank-order statistics; therefore, it can use various types of data, including chromatin accessibility and ChIP-seq. Enhancer-gene maps generated by these methods require sorting or purification methods to achieve cell type-specific maps.

Single-cell or single-nuclei ATAC-seq and RNA-seq allow for profiling all cell types and states across heterogeneous tissues. Methods like Cicero^104^, ArchR^105^, and DIRECT-NET^106^ utilize a meta-cell aggregation approach combining the nearest neighbor cells within cell states. Cicero utilizes co-accessibility network analysis to correlate accessibility signals aggregated from several cells at a time between pairs of genomic regions in a population of cells, penalizing for distance using a graphical LASSO and maximum interaction constraint of 500 kb^104^. ArchR calculates the Pearson correlation between normalized gene expression and peak accessibility across the meta-cells^105^. DIRECT-NET employs the XGBoost gradient-boosting model to regress gene expression against all candidate enhancers across meta-cells and then uses importance scores from XGBoost to identify functional enhancers^106^. SCENIC+ also utilizes gradient-boosting machine regression, GRNBoost2^107^, to estimate the importance of candidate enhancers for target gene expression, categorizing the results into positive and negative correlations^108^. SnapATAC^109^ utilizes a logistic regression model fit for each pair of binarized enhancer accessibility and gene expression. Finally, Signac^110^ and FigR^111^ take similar approaches using either Pearson or Spearman, respectively, correlations between gene expression and enhancer accessibility for each enhancer-gene pair. Both models select peaks (Signac = 200 peaks; FigR = 100 peaks) with similar GC content and total accessibility as background sets and calculate correlations between the target gene and these background peaks. A z-score is computed for each peak, and *p* values are computed using a one-sided *z*-test.

## Approaches evaluating functional effects of non-coding variation

Approximately 90% of GWAS variants are located in non-coding regions of the genome, with 30% of AD-associated variants estimated to fall within enhancer regulatory elements^34,40,112,113^. It is likely that these disease-associated variants disrupt regulatory element function, through mechanisms like transcription factor binding motif disruption, leading to dysregulation of gene expression. These variants are predicted to affect disease risk through small effects, and it may be the interaction of multiple variants that pushes the burden past the threshold to present phenotypically as a disease. Therefore, it is critical to functionally evaluate the effect of a variant in the relevant biological context.

Traditionally, reporter assays have been utilized to test the functionality of a candidate regulatory sequence. These assays test a sequence’s ability to increase the transcription of a reporter gene (GFP, LacZ, or luciferase)^114^. Massively parallel reporter assays (MPRAs) allow for the measurement of enhancer activity of thousands of candidate regulatory regions in a single experiment. A major advantage of MPRAs is the ability to synthesize sequences to be tested, allowing for straightforward testing of the effect of variation in these regions on enhancer activity using high-throughput sequencing^114,115^. MPRAs typically utilize episomes, taking the sequence out of its endogenous genomic context. On one hand, this is a strength in that the properties of a variant are tested completely independently; however, it is also a weakness because the observed properties may have reduced relevance when native context is restored^40^. Nevertheless, when performed in relevant models, MPRAs offer a great advancement in testing a high number of variants before proceeding to more involved functional experiments. For example, Bond et al.^116^ performed an MPRA of 3576 AD-associated variants in resting and proinflammatory macrophages and identified 47 expression-modulating variants (emVars), of which 39 were connected to 76 putative AD-risk genes through enhancer-promoter ABC pairs or microglia expression quantitative trait loci (eQTLs).

While MPRAs enable high-throughput screening for sequences able to increase transcription of a reporter gene, these assays provide no indication of the target gene of an enhancer. Many derivatives of CRISPR/Cas9 technologies have greatly improved our ability to functionally link CREs to target genes. First, CRISPR-based enhancer screens for genome perturbations have emerged in an effort to both determine the necessity of a sequence for a gene’s expression and link an enhancer to its target promoter. These methods utilize either an active Cas9 protein for sequence disruption or a catalytically inactive, nuclease dead Cas9 (dCas9) protein tethered to a repressor or activator domain^40,117–119^. The results of these perturbations are then determined by measuring gene expression, functionally linking enhancers to their target genes at scale. Second, traditional CRISPR experiments utilizing catalytically active Cas9 are considered the gold standard for verifying the effects of genetic variation. Nott et al. functionally validated a *BIN1* microglia-specific enhancer using CRISPR/Cas9-mediated deletion of the region in human pluripotent stem cells (PSCs). Gene expression analysis of microglia, neurons, and astrocytes from these PSCs confirmed that deletion of this enhancer region only affected *BIN1* expression in microglia and had no significant effects on expression in neurons and astrocytes^87^. However, one limitation to this finding is that the entire enhancer region was deleted (363 bp), meaning the SNV cannot be definitively assigned as causal. Approaches for making single-base pair edits are still the gold standard for causal variant validation in this regard. To this end, advancements in both iPSC-differentiation strategies and single-base pair editing through CRISPR/Cas9-based genetic engineering have improved the ability to make and model disease variants in relevant cell types. The iPSC neurodegenerative disease initiative (iNDI) has deeply characterized a single parental iPSC line and has created isogenic lines for more than 130 variants across 73 ADRD genes (8 AD, 15 DLB/PDD, 29 FTD/ALS, and 21 other)^120,121^. Further, improvements to prime editing have greatly increased the feasibility of testing variants of interest. Briefly, prime editing employs a Cas9 nickase, creating a single-strand DNA nick, and a specialized prime editing guide RNA (pegRNA) that both directs the Cas9 to the target site and encodes the desired genetic edit. A reverse transcriptase enzyme attached to the Cas9 nickase uses the pegRNA template to synthesize and insert the new DNA sequence directly at the target site, without requiring double-strand breaks or donor DNA templates^122^. This approach allows for highly precise and versatile edits, as well as being readily adaptable for use in iPSCs, which can be subsequently differentiated into disease-relevant cell types for downstream applications. Recently, Davis et al. performed prime editing in several mouse organs, including the brain, and achieved therapeutically relevant levels of successful prime editing (42% in the brain)^123^. This advancement could greatly improve gene therapies for neurodegenerative diseases in correcting harmful mutations in targeted tissue types.

### Future directions

Currently, the majority of data on disease-associated non-coding variation has largely been collected from populations of European ancestry, resulting in insufficient ancestral diversity and limiting our understanding of genetic contributors in underrepresented populations. This lack of representation is especially limiting given that African Americans and Hispanic Americans are more likely to develop AD than non-Hispanic whites from the same community^124^. Additionally, GWAS focused on African American and Japanese populations, while smaller than European population-focused studies, have identified novel loci not observed in European populations^125–127^. By continuing to diversify and increase the sample sizes of GWASs through efforts of large consortia like ReDLat^128^, LARGE-PD^129^, and GP2^21^ in AD and related dementias and PD, respectively, we can identify variants that are enriched in or specific to certain ancestries. These variants could then be incorporated into functional genomics experiments, like MPRAs and CRISPRi, to assess their effect on regulatory elements and expression of the target gene. Additionally, performing these experiments in cell lines derived from individuals of non-European ancestry allows for further assessment of the potential differences in genetic architecture at these disease-associated loci that may be mediated by trans-acting factors. Moreover, increasing the diversity of our current genomic studies enhances our understanding of AD etiology and improves the potential for success with genomic medicine interventions.

Targeting enhancers represents a promising therapeutic avenue for neurodegenerative diseases by fine-tuning gene expression at the regulatory level. For example, enhancers function through TF binding, so disrupting binding of TFs at enhancers associated with disease through small molecule inhibitors is a logistically difficult but potentially promising method for therapies. Another method for therapeutic intervention would be targeting eRNAs at disease-associated enhancers for degradation through antisense oligonucleotides^130^.

## Conclusions

GWAS and rare variant burden analyses have identified many loci associated with dementia, and prioritization strategies have pointed us to candidate causal variants to experimentally determine their functional impact. Chromatin conformation assays such as HiC and its derivatives have further refined our ability to identify enhancer-promoter pairs, linking CREs to their target genes with greater confidence. Similarly, single-cell and single-nucleus RNA-seq and ATAC-seq have revolutionized our understanding of cell type-specific gene regulation in disease-relevant brain cell populations, including neurons, astrocytes, and microglia. Advances in MPRAs and CRISPR-based functional screens have provided critical insights into how non-coding variants affect CRE activity and transcriptional regulation in cell type-specific and context-dependent manners. Additionally, the integration of multiomics datasets across diverse populations and disease stages offers an unprecedented opportunity to study both shared and distinct regulatory mechanisms underlying neurodegeneration. By combining data from these large-scale efforts across many samples, tissues, and cell types, we can gain a deeper understanding of dementia pathogenesis, uncovering novel therapeutic targets for these devastating neurodegenerative disorders.

## Data Availability

No datasets were generated or analysed during the current study.
